# Discovering PETases: An Interlink Between Engineering Enzymes and Microbiomes

**DOI:** 10.1111/1462-2920.70272

**Published:** 2026-03-11

**Authors:** Diego Javier Jiménez, Alexandre Soares Rosado

**Affiliations:** ^1^ Biological and Environmental Sciences and Engineering Division (BESE) King Abdullah University of Science and Technology (KAUST) Thuwal Saudi Arabia

**Keywords:** biocatalysis, metagenomics, microbiome perturbation, plastic degradation, polyethylene terephthalate, terrestrial ecosystem

## Abstract

Polyethylene terephthalate (PET), an abundant synthetic polyester, is the only plastic that has been enzymatically recycled at an industrial scale. Over the last decades, research efforts have focused on screening and engineering PET‐degrading hydrolases (PETases), aiming to identify variants that can operate efficiently in both environmental and industrial settings. The detection of potential PETases from marine and terrestrial ecosystems has primarily been conducted via metagenomics using homology strategies. However, the use of benchmark PETases as references has limited the searches, narrowing the sequence landscape. Currently, there remains a need to identify efficient thermophilic, halotolerant and pH‐robust PETases for the industrial biocatalysis of PET. In line with this, in this article, we discuss recent findings related to the following topics: (i) the identification of suitable ecosystems for mining PETases; (ii) the discovery of PETases via the restructuring of microbiomes; (iii) advancements in metagenomics and artificial intelligence (AI)‐based approaches for the detection and ranking of PETases and (iv) the future of PET biocatalysis. Overall, we suggest that disrupting microbiomes with polyester‐rich substrates, combined with innovative computational and AI‐based strategies, can be an effective pathway for the discovery of PETases that can be used as scaffolds for protein engineering and biotechnological applications.

## Graphical abstract

In this review‐type article, we discuss some recent trends in the identification of polyethylene terephthalate‐degrading enzymes (PETases) from nature. We highlight that the discovery of novel PETases can be improved by an interlink between reshaping microbiomes and engineering proteins.

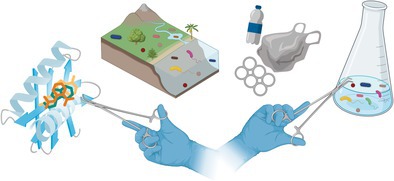

## 1 Introduction

Recent diplomatic efforts and international negotiations designed to regulate the global production and management of plastics have been unfruitful. At the present, pollution with synthetic plastics remains a pressing environmental concern, with large volumes of macro and microplastics continuing to accumulate in terrestrial and marine ecosystems, affecting wildlife, food security, human health and microbiomes (Zhu et al. 2025; Jiménez et al. 2022; Lear et al. 2021). A particularly abundant plastic waste is polyethylene terephthalate (PET), a polyester‐type thermoplastic commonly used to manufacture bottles, food containers and textile fibres. PET is a durable synthetic polymer that contains both crystalline regions, which confer structural stability, and amorphous sites, which allow it to be enzymatically depolymerised into its constitutive monomers (terephthalic acid [TPA] and ethylene glycol [EG]) mainly through the action of two hydrolases (PETases and MHETase) (Zimmermann 2025a).

The enzymatic conversion of PET is a process that has been well‐described for *Piscinibacter sakaiensis* (formerly *Ideonella sakaiensis*) (Yoshida et al. 2016). PETases are mostly found in taxa within the phyla *Pseudomonadota*, *Actinomycetota* and *Bacillota* (Danso et al. 2018; Pérez‐García et al. 2025), and they are divided into three main groups (I, IIa and IIb) based on their sequence similarity (Joo et al. 2018). Recently, a fourth group of PETases (type III) that includes halophilic enzymes (*Halo*PETase1 and dsPETase05) found in ocean‐derived metagenomes has been proposed (Turak et al. 2025). A key feature of most PETases is their alpha/beta‐fold structure, though it has been demonstrated that other enzymes with different folds can also depolymerise PET (Erickson et al. 2022). The native substrate of most PETases is cutin, a polyester‐rich compound found in plant cell walls, but these enzymes are highly promiscuous, with broad substrate spectra and low substrate specificity (Zhang et al. 2024; Ahituv et al. 2025). Due to their promiscuity, PETases can depolymerise other types of plastic, such as polybutylene adipate terephthalate (PBAT), polylactic acid (PLA), polycaprolactone (PCL) and polyurethane (PUR) (Zimmermann 2025a). PET‐active enzymes have been retrieved in various microbial systems, such as in hot springs (Erickson et al. 2022), glaciers (Qi et al. 2023), compost (Sulaiman et al. 2012), the deep sea (Chen et al. 2024), human saliva (White and Wallace 2023) and oceans (Alam et al. 2025).

Currently, PET is the only synthetic plastic that is enzymatically recyclable on an industrial scale (TRL9) within a circular bioeconomy framework (Zimmermann 2025a, 2025b; Wei, Weber, et al. 2025a). In industrial scenarios, a pretreatment aimed to reduce the percentage of crystallinity of PET (known as amorphisation) is needed. In addition, high temperatures (~70°C) increase the flexibility of PET, improving access for enzymatic attack and the efficiency of depolymerisation until gradual conversion of amorphous fraction with subsequent increases in crystallinity (Wei, Weber, et al. 2025a; Zimmermann 2025a, 2025b; Wei et al. 2019). Over the last decade, wild‐type PETases have been engineered to generate thermotolerant variants (e.g., PES‐H1^L92F/Q94Y^, HotPETase and LCC^ICCG^) (Arnal et al. 2023; Bell et al. 2022). Other engineered enzymes such as TurboPETase can achieve nearly complete depolymerisation in 8 h at a solid loading of 200 g/kg, with its improved performance attributed to a flexible PET‐binding groove (Cui et al. 2024). However, to date, only one engineered thermotolerant PETase (LCC^ICCG^) has been efficiently used to depolymerise PET in industrial conditions (Arnal et al. 2023). This has been done by the company Carbios that expect to move forward with a PET enzymatic recycling facility in the north of France. Unfortunately, the efficient depolymerisation of PET remains challenging and expensive due to the downstream steps involved. For example, within the reactors, a high PET loading leads to the accumulation of TPA and consequently a pH reduction, which requires the addition of high concentrations of salts (e.g., NaOH) for neutralisation. Thus, PETases with activity at high temperatures, at a low pH, towards crystalline PET, at high PET loads, and in saline conditions are required to optimise catalytic processes (Wei, Weber, et al. 2025a; Colizzi et al. 2025; Groseclose and Nguyen 2025). In this context, the discovery, characterisation and engineering of novel thermophilic, acidophilic and halophilic PET‐transforming enzymes have emerged as important topics of research (Mican et al. 2024; Wei, Westh, et al. 2025b; Zhang et al. 2025; Groseclose and Nguyen 2025).

The success of PETase engineering has led to underestimating the potential of environmental microbiomes as a source of novel and efficient enzymes. In this review, we discuss recent discoveries within this topic, highlighting strategies used to improve the capture of PETases in natural ecosystems. In doing so, we propose a conceptual framework encompassing targeted ecosystem mining, deliberate microbiome reshaping, gene‐centric metagenomics and artificial intelligence (AI)‐assisted analysis to expand the sequence/structure space beyond known PETase families. This manuscript differs from prior reviews and/or perspective‐type articles (Viljakainen and Hug 2021; Jiménez et al. 2022; Mican et al. 2024; Sun 2024; He et al. 2025; Wei, Westh, et al. 2025b; Pérez‐García et al. 2025; Zimmermann 2025b; Ahituv et al. 2025) by foregrounding microbial diversity and ecological selection. Particularly, four main questions are addressed:

1. Which environmental microbiomes hold the greatest potential in the search for effective PETases?
2. Which strategies can facilitate the discovery of novel PETases in these microbiomes?
3. How can metagenomics and AI‐based tools support the detection and ranking of predictive PETases?
4. What is the future for the bio‐based transformation of PET?

## 2 Well‐Suited Microbial Sources of PET‐Active Enzymes

In the last two decades, several studies and metagenomic surveys have focused on identifying PET‐active enzymes in distinct natural ecosystems (Danso et al. 2018; Erickson et al. 2022; Alam et al. 2025). For example, the wild‐type version of LCC^ICCG^ was originally obtained from a leaf‐branch compost metagenome (Sulaiman et al. 2012). Some studies have reported that terrestrial microbiomes contain a higher occurrence of putative PETases and an expanded potential to degrade polyesters compared to marine microbiomes (Danso et al. 2018; Contreras‐Moll et al. 2025). In addition, a clear taxonomic pattern has been observed in which predicted PETases from *Actinobacteriota* species are more common in terrestrial ecosystems, whereas putative PETases from *Pseudomonadota* and *Bacteroidota* are frequently found in marine environments (Danso et al. 2018; Alam et al. 2025). However, with the advancement of sequencing and the increase of metagenome data, these taxonomic trends should be tested using recently compiled global gene catalogues (Coelho et al. 2022) with common thresholds and standardised datasets.

**FIGURE 1:**
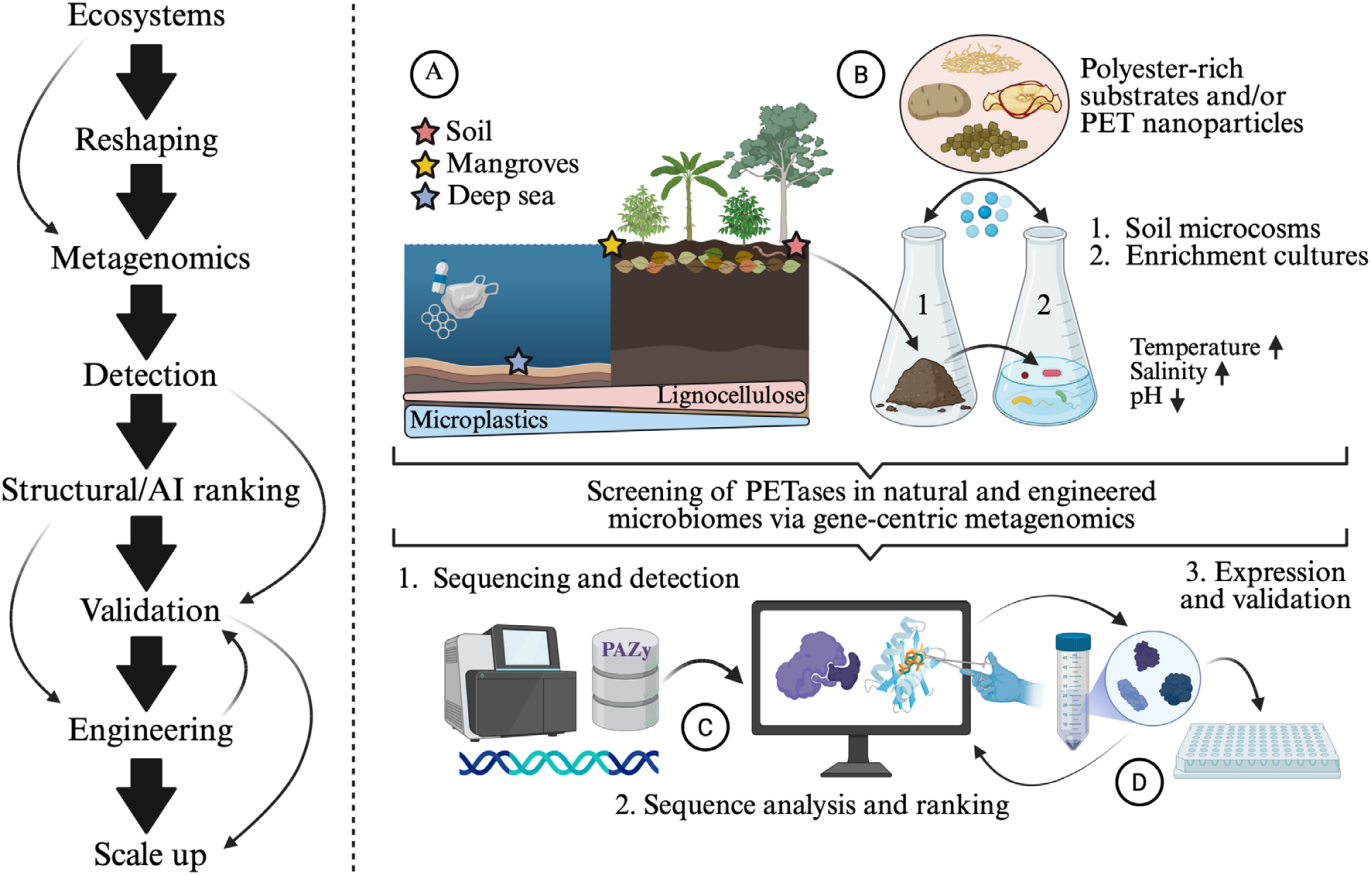
Conceptual roadmap and key topics associated with the detection of PET‐active enzymes from microbiomes. Left, an iterative process flow, representing the major steps and feedback loops involved in the discovery, characterisation and testing of PETases. (A) Presents three well‐suited ecosystems for the search for PETases, indicating the prevalence of microplastics and lignocellulose. (B) Shows two interconnected selective wet‐lab strategies (i.e., soil microcosm and enrichment culture experiments) used to restructure microbiomes, aiming of improving the prevalence and detection of polyester‐degrading microbial taxa and enzymes. Polyester‐rich substrates, PET nanoparticles and environmental control can be used to select for thermophilic, acidophilic and/or halophilic microbial communities with potential PET‐degrading capabilities. (C) Presents the construction of full gene catalogues from metagenomes and the screening of PETases using specialised and curated databases such as PAZy. The in‐depth sequence analysis of candidate PETases is necessary to identify key catalytic residues, disulphide bond positions, 3D structural traits and to predict enzyme features (e.g., solubility, melting temperature and pH) via AI‐based tools. (D) Presents a feedback process in which candidate PETases are produced in a heterologous host, tested in high‐throughput enzymatic assays (see Box 1) and engineered to improve their efficiency under harsh environmental conditions.

Recent studies suggest that *Halopseudomonas* species are a major microbial source of halophilic PETases within ocean‐derived microbiomes (Turak et al. 2025; Alam et al. 2025; Chen et al. 2024). However, *Marinobacter*, *Vibrio*, *Nocardioides* and *Ketobacter* species have also been identified as alternative sources of novel PETases within marine ecosystems (Meyer‐Cifuentes et al. 2020; Rathod and Biswas 2026; Weigert et al. 2022). Species belonging to the *Planctomycetota* phylum are known to degrade polysaccharides in marine environments (Klimek et al. 2024), and they may contain several yet‐unknown polyester hydrolases with potential activity against PET, but this possibility remains overlooked. In addition, deep‐sea ecosystems (e.g., hydrothermal vents), which are sinks for microplastics, appear to be a promising source of halotolerant and thermotolerant PETases (Figure 1A) (Chen et al. 2024). For example, a well‐characterised PET‐active enzyme (a feruloyl esterase, PET46) found within the *Archaea* domain was identified within a metagenome‐assembled genome (MAG) derived from Guaymas Basin hydrothermal sediment (Perez‐Garcia et al. 2023).

The high prevalence of predictive PETases in terrestrial microbiomes is potentially associated with the continuous input of plant polymers (e.g., cutin, suberin and xylan‐rich substrates) that contain the same ester linkages found in PET (Chen et al. 2020; Cai et al. 2025). Thus, terrestrial ecosystems containing natural polyesters that are analogues to PET (e.g., beeswax, cork, potato epidermis, aquatic plants and fruit cuticles) are well‐suited for the search of PET‐active taxa and enzymes (Cai et al. 2025). In addition, man‐made systems with a substantial input of organic matter and plastics (e.g., garbage dumps, compost and landfills) are reported as a source of plastic‐degrading enzymes (Lin et al. 2025). For example, *P. sakaiensis*, a PET‐degrading bacterial model, was isolated from an enrichment culture containing PET‐contaminated sediment (Yoshida et al. 2016). Mangrove soils are ideal ecosystems to search for bacterial and archaeal halophilic PETases because their associated microbiomes have a complex diversity (Jiménez, Chaparro, et al. 2025) and are constantly exposed to plant polymers (via plant litter) and plastic pollution (Deakin et al. 2025) (Figure 1A). However, to date, there have been no reports of active PET‐degrading enzymes derived from mangrove‐associated microbiomes.

Host‐associated microbiomes in which plant biomass transformation processes frequently occur have also been reported as a source of polyester‐degrading enzymes. For example, Quartinello et al. (2021) demonstrated that cow rumen fluids can hydrolyse synthetic polyester, while Mamtimin et al. (2024) described a novel PETase (a feruloyl esterase) from the gut microbiome of mealworm ( *Tenebrio molitor* ) larvae. Notably, a recent study reported that fluids from carnivorous plants (*Nepenthes* spp.) are a source of proteases that can transform polyesters. These interesting findings raise questions about the role and use of proteases in PET depolymerisation processes (Siracusa et al. 2025) and suggest that non‐canonical hydrolases, including some proteases and esterases, may contribute to PET transformation in nature.

## 3 Restructuring Environmental Microbiomes to Uncover Novel PET‐Degrading Capabilities

Predictive PETases are considered rare enzymes because their prevalence in environmental microbiomes is very low, with less than 1 hit per Mbp of metagenome information in terrestrial ecosystems (Danso et al. 2018). Thus, the artificial and controlled restructuring of environmental microbiomes via microcosms or enrichment cultures has the potential to increase the likelihood of identifying novel PETases (Jiménez et al. 2024; Jiménez, Jamil, et al. 2025). In other words, by altering the structure of an environmental microbiome, the existing microbial hierarchy can be disrupted, leading to the selection of target or rare populations (Delmont et al. 2015) with potential PET‐depolymerising capabilities.

Recently, static microcosm experiments have been designed with deep‐sea sediments amended with PET microparticles (Zhao et al. 2023) and mangrove sediments amended with PET films (Saidu et al. 2025) as an approach to select and enrich PET‐degrading prokaryotic species. Although the use of amorphous PET to promote this selection process is a rational strategy, the inclusion of natural PET analogue substrates or plant biomass (e.g., agricultural residues, cork, potato epidermis and fruit cuticles) to soil or sediment microcosms can drastically restructure their microbial communities, thus enriching potential PET‐depolymerising taxa and enzymes (Figure 1B). In line with this, a recent study by our team reported that the addition of rice husk to mangrove soil microcosms increased the prevalence of putative halophilic PETases in the corresponding metagenomes, with some of these enzymes affiliated with *Marinobacter* and *Microbulbifier* species (Peña‐Valencia et al. 2025).

Recent studies have suggested that the transformation of PET and its monomers is more effective when performed by microbial consortia rather than single microorganisms (Bao et al. 2023; He et al. 2025). This occurs because the presence of specialists and generalists, enzymatic synergism and division of labour is common in polyester‐degrading microbial consortia (Schaerer et al. 2023; Meyer‐Cifuentes et al. 2020; Liu et al. 2025; Jiménez, Jamil, et al. 2025). To assess the efficiency of PET degradation some synthetic microbial consortia (SynComs) have been assembled, with *Pseudomonas* and *Bacillus* species emerging as key members of these PET‐degrading SynComs (Roberts et al. 2020; Qi et al. 2021; Salinas et al. 2024). Moreover, the selection of PET‐transforming microbial consortia via liquid enrichment cultures (i.e., in a top‐down approach) has the potential to assist in clarifying the ecology and enzymology associated with the degradation of PET. It also offers a pathway to improve the discovery of novel taxa and enzymes involved in this metabolic process (Viljakainen and Hug 2021).

Top‐down enrichment approaches have been applied to an array of environmental microbiomes, including plastic‐polluted soils (Roman et al. 2024) and plastic‐associated biofilms (Howard et al. 2023). Recently, an enrichment strategy to artificially select PET‐transforming microbial consortia from mangrove soil microcosms has been developed (Jiménez, Jamil, et al. 2025). In this process, the modification of culture conditions along growth‐dilution cycles enhanced the microbial cell density in a minimal liquid media containing PET as the sole carbon source. Genome‐centric metagenomic analysis in the resulting microbial consortia allowed the identification of novel taxa (e.g., *Mangrovimarina plasticivorans*) and predictive PET‐transforming enzymes, including enzymes involved in TPA and EG catabolism (Jiménez, Jamil, et al. 2025). However, metasecretomic analyses are required to identify putative PETases that are secreted during the growth of selected microbial consortia, as has been reported for lignocellulose‐ and PBAT‐degrading microbial consortia (Jiménez et al. 2015; Meyer‐Cifuentes et al. 2020).

In the selection of microbial consortia with PET‐degrading capabilities, different substrates can be used as the sole source of carbon instead of bulk PET, such as PET nanoparticles, natural polyester‐rich substrates (i.e., PET analogues) or lignocellulose (Jiménez et al. 2024). The use of PET nanoparticles could favour microbial growth due to their higher surface‐to‐volume ratio and lower molecular weight compared to bulk PET. Moreover, starting with microbial communities that have pre‐adapted to natural polyester‐rich substrates may increase the chances of selecting putative PET‐degrading taxa and enzymes within liquid enrichment cultures (Figure 1B). This assumption is based on the historical contingencies or legacy effects reported in the selection of lignocellulose‐degrading communities (Jiménez et al. 2017; Kalenitchenko et al. 2021). Microbial consortia that thrive on lignocellulose have the potential to be source of PETases. For example, 27 predictive PETases were detected from 20 genomes in an Andean soil‐derived lignocellulolytic microbial consortium. Two of these putative PETases were found in the genome of a novel bacterial taxa (*Andeanibacterium colombiense*), with one of these flanked by a tannase/feruloyl esterase, which is part of the same family of MHETases (Díaz‐García et al. 2024; Knott et al. 2020). Notably, a mutated tannase/feruloyl esterase from *Fusarium oxysporum* has shown activity on MHET and lignocellulose (Makryniotis et al. 2025).

## 4 Screening and Ranking of Predictive PET‐Degrading Enzymes in Metagenomes

Metagenomes can be analysed in multidimensional ways (e.g., from reads to genomes) (Peng et al. 2025). However, to minimise the loss of information and maximise candidate recovery, the assembly of reads into contigs, followed by the development of full gene catalogues is the optimal approach for the bioprospection of PETases from metagenomes. Since the discovery of LCC (Sulaiman et al. 2012) and *Is*PETase (Yoshida et al. 2016), several studies have focused on the exploration of metagenomes to screen PETases (Danso et al. 2018; Erickson et al. 2022; Chen et al. 2024; Seo et al. 2025). Current approaches are based on sequence similarity, including multiple sequence alignments (e.g., BLAST) or hidden Markov model (HMM) searches (Chow et al. 2023) against known PET‐active enzymes deposited in specific databases, such as PAZy (Buchholz et al. 2022) or PlasticDB (Gambarini et al. 2022) (Figure 1C). HMM searches appear to be more sensitive, while BLAST may generate false‐positive results, detecting alpha/beta‐fold hydrolases with low or not activity on PET (known as pseudo‐PETases). Thus, different sequence homology thresholds are advocated to be used, allowing to identify enzymes with moderate (≧ 50% identity and ≧ 80% coverage) to high (≧ 70% identity and ≧ 90% coverage) similarity with known PET‐active enzymes (Peña‐Valencia et al. 2025). While HMM scores of ≧ 100 indicate confident homology, enzymes with an HMM score higher than 70 have also shown enzymatic activity on PET (Erickson et al. 2022). Overall, these approaches are highly restricted to the known sequence space of PETases (Mican et al. 2024).

Recently, a similarity‐based search for predictive PET‐active enzymes in expanded public global assemblies was reported, generating around 215 million non‐redundant PAZy homologues (Chikhi et al. 2024). These sequences are publicly accessible in the PETadex dataset. Another strategy to identify PETases in gene catalogues is searching for specific protein signatures. For example, the functional M5 motif has been used to identify PETases and pseudo‐PETases in catalogues derived from ocean metagenomes (Alam et al. 2025). However, this strategy is limited because several novel PET‐active enzymes lack this motif, including GlacPETase (Qi et al. 2023), *Halo*PETase1 (Turak et al. 2025) and 101 (Erickson et al. 2022).

An interesting methodology that combines protein network analysis and enzymatic activity assays was recently used to detect and rank PETases from the polyesterase‐lipase‐cutinase family. Based on this, a set of proteins was selected, engineered and compared with benchmark enzymes (Seo et al. 2025). However, innovative computational pipelines are still required, such as using 3D structural traits to screen for potential PETases in metagenomes (Robinson 2023). Therefore, similarity‐based searches must be compared for consensus, and they need to be complemented with 3D structural analysis and molecular docking to detect catalytic regions, disulphide bond sites, particular structural confirmations, and key residues involved in PET turnover (Mican et al. 2024; Peña‐Valencia et al. 2025). This topic is progressing with the implementation of new AI‐based tools and protein embeddings for classification (Jahanshahi et al. 2025). Machine learning (ML)‐based frameworks have been designed to predict the ability of an enzyme to degrade plastics (Jiang et al. 2023) and to identify plastic‐degrading enzymes in a public protein dataset (Jiang et al. 2024). In addition, an interesting ML‐based approach (Yu et al. 2023) was implemented to identify putative plastic‐degrading enzymes in a global collection of metagenomes from landfill sites (Lin et al. 2025).

A computational workflow for the discovery, enrichment, filtering, ranking and refinement of candidate PETases was recently proposed by Mican et al. (2024), but the genomic context of known PET‐active enzymes (e.g., *Is*PETase, LCC and *Halo*PETase1) was not considered in this workflow and it is still underexplored. This information could help to guide the mining of novel PETases, as is used to improve the annotation of genes with unknown functions in metagenomes (Rodríguez Del Río et al. 2024). Moreover, 3D protein structures, kinetic parameters (e.g., *k*_cat_ and *K*_m_), optimal conditions (e.g., pH, salinity and temperature), solubility and the melting temperature (*T*_m_) can be predicted using AI‐based integrative pipelines, for example as ProtScout (Peña‐Valencia et al. 2025). Additionally, sequence and structural analysis, including molecular docking, has been used to mine and prioritise a specific set of alpha/beta‐fold hydrolases (Vasina et al. 2022). This multifaceted approach produces a reliable ranking of PETases, accelerating the discovery process and helping to detect the most promising candidates for further activity testing (see Box 1) or protein engineering trials (Figure 1C,D). It is also predicted that the use of quantum computing approaches can accelerate the discovery and engineering of PETases (Damborský et al. 2025).

BOX 1 **Expression and Activity Assays for PET‐Degrading Enzymes.**

The screening of PETases in environmental metagenomes can yield hundreds to millions of putative active enzymes (Danso et al. 2018; Seo et al. 2025; Chikhi et al. 2024). However, most of them are likely to be pseudo‐PETases or enzymes that are only active on PET oligomers. Thus, it is crucial to identify proteins with high activity on PET polymer for subsequent enzymatic characterisation. Production of predictive PETases in a heterologous host requires the removal of signal peptides from selected enzymes, gene codon optimisation, chemical synthesis, cloning, expression and protein purification. A common bottleneck in this process is the formation of inclusion bodies when the putative PETases are expressed in *E. coli* , which can hinder enzymatic activity tests (Mican et al. 2024). Additionally, an appropriate protein expression system is necessary to ensure the formation of disulphide bonds.

Once putative PETases are produced, their activity can be assessed using different methods. Recently, Yew et al. (2025) have discussed the benefits and drawbacks of high‐throughput strategies for the testing of PETase activity. Microtiter plate assays with PET nanoparticles, PET coatings or PET‐analogue chromogenic substrates appeared to be the most versatile platforms (Figure 1D). These methods are frequently combined with spectrophotometric analysis and/or the detection of PET monomers using high‐performance liquid chromatography (Weigert et al. 2021). The esterase activity of PETases is typically evaluated using chromogenic substrates, such as *para*‐nitrophenyl butyrate. However, esterase activity on *p*‐nitrophenyl fatty acid esters does not correlate with activity on PET or other polyesters (Makryniotis et al. 2024). Recently, researchers have designed new labelled compounds containing chromo‐ or fluorogenic moieties to mimic the structure of PET and enable the real‐time monitoring of enzyme activity and kinetics (Taxeidis et al. 2024). However, these substrates are synthetic and activity against post‐consumer PET or PET films is desired. Nevertheless, this field is advancing rapidly with the development of novel, less expensive and more facile methods, including the use of impedance, changes in pH and PET nanoparticles with dyes. However, standardised methods and procedures are still needed for valid comparisons (Colizzi et al. 2025; Wei, Westh, et al. 2025b).

## 5 The Future and Beyond of the Enzymatic Transformation of PET

Searching for PET‐active enzymes in different ecosystems and microbial lineages is hot topic. However, the phylum *Archaea* is still underexplored; it may harbour several PETases that are active under polyextremophilic conditions (e.g., low pH, high temperatures or high salinity). For example, thermo‐acidophilic archaeal species with the potential to deconstruct polysaccharides (Prokofeva et al. 2025) can be a source of hydrolases with PET activity. To date, only four PET‐active enzymes with an archaeal origin have been reported (Pérez‐García et al. 2025). Other extremophiles, such as *Deinococcus maricopenis* (isolated from desert soil), have demonstrated to be source of novel polyesterases with activity on PET and comparable to LCC^ICCG^ (Makryniotis et al. 2023). As mentioned in the introduction, a new group of bacterial PETases has been reported (Turak et al. 2025), and it is predicted that other families will be described in the coming years (Peña‐Valencia et al. 2025). Many of these may adopt noncanonical 3D folds with a distinct 3D protein structure. Thus, exploring folds other than the alpha/beta‐fold is suggested for non‐natural substrates such as PET (Mican et al. 2024; Colizzi et al. 2025). In terms of catalytic properties, mining PETases from natural ecosystems may be more successful than engineering them, as has been reported for other alpha/beta‐fold enzyme classes (Vasina et al. 2022).

Many researchers worldwide are searching for novel PETases that outperform the efficiency of LCC and its variants (e.g., LCC^ICCG^). In recent years, proteins from different families of PETase have been engineered to improve their activity at high temperatures (Lu et al. 2022; Blázquez‐Sánchez et al. 2023; Wang et al. 2025; Zhang et al. 2025). Moreover, recent tournaments have been created to improve the activity of known PET‐active scaffolds, aiming to find new benchmark variants that can be more efficient. New AI‐based approaches will become more relevant in the design of new variants of existing PETases (Lu et al. 2022; Joho et al. 2024). In addition, it is predicted that the future of enzymatic PET depolymerisation lies in the *de novo* creation of minimal protein scaffolds with improved features, as reported for polycarbonate‐degrading enzymes (Holst et al. 2023).

The design, testing and use of enzymatic cocktails in PET depolymerisation processes in industrial settings have both advantages and disadvantages. For example, the use of a mixture of different PETase variants or dual‐enzyme systems (e.g., PETase–MHETase) can increase the rates of depolymerisation, decrease the enzyme inhibition effects due to PET oligomers, and enhance the release of PET‐derived monomers (Knott et al. 2020; Zhang et al. 2023; Mabashi‐Asazuma et al. 2025). However, it is also predicted that the use of enzyme cocktails will increase operating costs in scaled up settings. Moreover, the use of alternative proteins to boost the depolymerisation of crystalline PET is advocated and necessary. To achieve this, lytic polysaccharide monooxygenases and hydrophobins are suitable candidates that can bind to PET, modifying its hydrophilicity and promoting subsequent enzymatic activity (Munzone et al. 2024; Corrêa et al. 2025).

Beyond the enzymatic transformation of polyesters, whole‐cell systems have been developed to transform PET. For example, *Pseudomonas umsongensis* , a microbe that can metabolise PET monomers, has been engineered to secrete an efficient PETase (PHL7) (Banks et al. 2024), similarly as is reported for *P. putida* (Brandenberg et al. 2022). Moreover, a broad‐host‐range conjugative plasmid has been used to transform wastewater‐derived microbial species, giving them the capacity to produce a PETase under certain conditions (Yip et al. 2024). In addition, an innovative strategy, called GenRewire, has been developed with the aim of producing microbial species with PET‐degrading capabilities via rewiring and genome editing without the inclusion of foreign DNA (Vidal et al. 2025). This approach can revolutionise the catalysis of plastics in industrial and natural bioremediation scenarios. Ultimately, however, efficient PET transformation and the development of novel enzymatic technologies will require close collaboration between microbiologists, computational biologists, polymer scientists and enzymologists (Jiménez et al. 2022; Colizzi et al. 2025).

## 6 Conclusions

Currently, sequence homology strategies used to screen PETases in environmental microbiomes are biased due to the references known PETases present in the databases. As a result, the detection of potential novel PETases is limited because searches are restricted to those that exhibit moderate to high sequence similarity with known and characterised PETases, such as *Is*PETase, LCC and PHL7. After reviewing recent literature, we propose that exploring terrestrial ecosystems, such as mangroves, in combination with microbiome perturbation using natural polyester‐rich substrates and innovative search strategies, including AI‐based methods and the incorporation of 3D protein structures, will enhance the discovery of novel putative PETases. This form of exploration should also be paired with structural analysis, enzymatic trait prediction and high‐throughput assessments of enzymatic PET activity. While protein engineering can lead to improved variants for industrial applications, microbiome engineering strategies have the potential to unveil novel taxa and enzymes with PET‐degrading capabilities. Additionally, these strategies can provide insights into the eco‐enzymology associated with PET transformation in natural environments. Furthermore, there is fierce competition to develop the most efficient engineered PETases for industrial enzymatic recycling. However, there are several challenges to consider in this process, including the crystallinity of PET, downstream processing and economic feasibility. Nature can probably provide improved PETases, but innovative strategies are required to identify them in a complex ‘jungle’ of microbial species. In some enzyme classes, nature‐derived scaffolds outperform heavily engineered variants, suggesting that mining natural microbial diversity may sometimes outpace exhaustive enzyme engineering. New methods, strategies and technologies are constantly being developed, but advancing the biocatalysis of PET requires collaboration between scientists, companies, institutions and governments, fuelled by the vast diversity and functionality hidden in environmental microbiomes.

## Author Contributions

Conceptualisation: Diego Javier Jiménez. Data curation: Diego Javier Jiménez. Formal analysis: Diego Javier Jiménez. Funding acquisition: Alexandre Soares Rosado. Investigation: Diego Javier Jiménez and Alexandre Soares Rosado. Visualisation: Diego Javier Jiménez. Writing – original draft: Diego Javier Jiménez. Writing – review and editing: Diego Javier Jiménez and Alexandre Soares Rosado.

## Funding

This work was supported by King Abdullah University of Science and Technology (BAS/1/1096‐01‐01).

## Conflicts of Interest

The authors declare no conflicts of interest.

## Data Availability

Data sharing not applicable to this article as no datasets were generated or analysed during the current study.
